# The effect of an active implementation of a disease management programme for chronic obstructive pulmonary disease on healthcare utilization - a cluster-randomised controlled trial

**DOI:** 10.1186/1472-6963-13-385

**Published:** 2013-10-03

**Authors:** Margrethe Smidth, Morten Bondo Christensen, Morten Fenger-Grøn, Frede Olesen, Peter Vedsted

**Affiliations:** 1 The Research Unit for General Practice, Aarhus University, Aarhus, Denmark; 2 The Section for General Medical Practice, Aarhus University, Aarhus, Denmark

**Keywords:** Implementation, Disease management, Healthcare utilization, COPD, Chronic Care Model, RCT

## Abstract

**Background:**

The growing population living with chronic conditions calls for efficient healthcare-planning and effective care. Implementing disease-management-programmes is one option for responding to this demand. Knowledge is scarce about the effect of implementation processes and their effect on patients; only few studies have reported the effectiveness of disease-management-programmes targeting patients with chronic obstructive pulmonary disease (COPD). The objective of this paper was to determine the effect on healthcare-utilization of an active implementation model for a disease-management-programme for patients with one of the major multimorbidity diseases, COPD.

**Methods:**

The standard implementation of a new disease-management-programme for COPD was ongoing during the study-period from November 2008 to November 2010 in the Central Denmark Region. We wanted to test a strategy using Breakthrough Series, academic detailing and lists of patients with COPD. It targeted GPs and three hospitals serving approx. 60,000 inhabitants aged 35 or older and included interventions directed at professionals, organisations and patients. The study was a non-blinded block- and cluster-randomised controlled trial with GP-practices as the unit of randomisation. In Ringkoebing-Skjern Municipality, Denmark, 16 GP-practices involving 38 GPs were randomised to either the intervention-group or the control-group. A comparable neighbouring municipality acted as an external-control-group which included nine GP-practices with 25 GPs. An algorithm based on health-registry-data on lung-related contacts to the healthcare-system identified 2,736 patients who were alive at the end of the study-period. The population included in this study counted 1,372 (69.2%) patients who responded to the baseline questionnaire and confirmed their COPD diagnosis; 458 (33.4%) patients were from the intervention-group, 376 (27.4%) from the control-group and 538(39.2%) from the external-control-group. The primary outcome was adherence to the disease-management-programme measured at patient-level by use of specific services from general practice. Secondary outcomes were use of out-of-hours-services, outpatient-clinic, and emergency-department and hospital-admissions.

**Results:**

The intervention practices provided more planned preventive consultations, additional preventive consultations and spirometries than non-intervention practices. A comparison of the development in the intervention practices with the development in the control-practices showed that the intervention resulted in more planned preventive-consultations, fewer conventional consultations and fewer patients admitted without a lung-related-diagnosis.

**Conclusions:**

Use of the active implementation model for the disease-management-programme for COPD changed the healthcare utilization in accordance with the programme.

**Trial registration:**

Clinicaltrials.gov identifier: NCT01228708.

## Background

The number of people living with chronic conditions is growing as a result of lifestyle, increased life-expectancy, improved treatment options and growing diagnostic activity [1]. This situation demands efficient healthcare planning and effective care. Danish healthcare is tax-financed and citizens therefore enjoy free use of healthcare at the point of care. The nearly 3,600 general practitioners (GPs) are gatekeepers for access to most of the healthcare system. The GPs operate as independent contractors under a system of five regions and are remunerated on a combination of fee-for-service and capitation basis (75/25). Each of the 98 Danish municipalities is responsible for providing rehabilitation services and preventive care for their citizens. The five Danish regions are responsible for services from hospitals and general practice.

The Danish Health and Medicines Authority estimates that 80% of healthcare costs are spent on people living with one or more chronic conditions [2]. It has been argued that healthcare may be improved and public spending reduced by implementation of an efficient strategy that ensures seamless care for patients through the concerted effort of general practices (GP practices), hospitals and the municipalities [3-6]. Only few studies have investigated the economy and the healthcare utilization of the integrated care programmes [7]. Wennberg has dismissed the idea that ”more is better” within healthcare provision [8], but argues that the care provided rather needs to be targeted and focused.

Chronic obstructive pulmonary disease (COPD) is an important non-communicable disease. It ranks as the fifth leading cause of death globally [9], and Denmark has the highest COPD incidence rate among the European countries [10]. COPD is an under-diagnosed, irreversible and potentially life-threatening condition where secondary prevention, treatment and rehabilitation with systematic follow-up may help control the symptoms, increase the patient’s quality of life and delay disease progression [11]. Newly published results indicate that 14.3% of people aged 35 years and older have COPD [12]; yet, only 28% of these people have been diagnosed [13].

In 2008, the Central Denmark Region implemented a new disease management programme for COPD inspired by the Chronic Care Model (CCM) [14]. However, implementation can be challenging, and knowledge about the effects of the implementation processes and their possible effect on patients remains scarce [15,16]. Lugtenberg et al. argue that active leadership is required to implement change [17], and Greenhalgh states that targeted change must be simple and adjustable to each locality [18]. Grol et al. show that implementation in healthcare needs to involve evidence-based information, few and precise recommendations and practical advice to secure implementation in clinical practice [19]. Several randomised studies have reported that implementation of disease management programmes for chronic conditions could reduce hospital admissions, out-of-hours services and emergency department attendance [20-24]. One cohort study found that implementation of a disease management programme reduced readmissions to hospital [25], but only few studies have examined the effectiveness of disease management programmes targeting patients with COPD [26,27].

The aims were, first, to investigate the impact of the previously developed active implementation model for a disease management programme for COPD as measured by specific indicators to determine to which degree the GPs follow the recommendations; and, second, to determine the extent of healthcare utilization in primary and secondary care for patients with COPD. The programme was developed on the basis of the CCM [28].

## Methods

### Study design

The study was a block- and cluster-randomised controlled trial with intervention and control groups and an additional external control group. The intervention group consisted of patients from half of the general practices in the Ringkoebing-Skjern Municipality in Denmark. Patients from the other half of the practices formed the control group. Patients in a comparable neighbouring municipality (Ikast-Brande) formed the external control group which was established to control for a spillover effect of the intervention conducted within the municipality [29,30]. To identify patients highly suspected for having COPD, we used a validated COPD algorithm [31].

### Setting

The study period spanned from November 2008 to December 2010. The study was conducted in the western part of the Central Denmark Region, where secondary care is provided by three regional hospitals in Ringkoebing, Herning and Holstebro. Two comparable municipalities in the region provided the setting for the study.

All citizens living in Denmark are registered with a personal identification number, the CPR number. This allows unique linkage between all the national registries at the level of the individual [32]. The GPs are eligible for reimbursement of the services they provide and they must submit electronic reimbursement claims to the Danish National Health Insurance Service Registry (DNHISR). Information about all Danish citizens’ use of general practice services, including out-of-hours services, can be obtained from the DNHISR [33,34]. All hospitals must report out-patient visits, contacts to the emergency department, hospital admissions and discharges to the regional Patient Administrative System (PAS) using International Classification of Diseases (ICD-10) codes.

During the study period, the Ringkoebing-Skjern Municipality had approximately 58,000 inhabitants of whom 35,000 were aged 35 or above. The municipality had 38 GPs organised into 15 practices. The neighbouring municipality (Ikast-Brande) had close to 40,000 inhabitants of whom 24,000 were aged 35 or above, and 25 GPs organised into ten practices. In all practices, staff (i.e. nurses, laboratory technicians or secretaries) employed by the GPs conducted either the whole or a part of the consultations on their own.

### Intervention

The intervention practices undertook an active, structured implementation of a disease management programme for COPD. The intervention is depicted in the Additional file 1; we have described the development of the intervention in detail elsewhere [35]. The intervention comprised components from the main areas of the CCM - Policies and Resources, Self-Management Support, Delivery System Design, Organisation of Healthcare and Clinical Information System [36]. To stimulate the process, we asked a local, esteemed opinion leader to introduce and support the intervention [37] both to GPs and to the municipality.

The intervention practices were invited to participate in four two-and-a-half-hour sessions. The Breakthrough Series [38,39] was used as a framework for the implementation of planned and targeted changes. All meetings were chaired by experts and experienced facilitators, who were all clinically educated and experienced in aiding change in practice. One facilitator (MS) visited each practice to explore and/or address challenges encountered in pursuing their goals.

We negotiated our implementation strategy with the municipality, which took active ownership by increasing the number of free COPD courses and smoking cessation courses. The region agreed on providing a special reimbursement to GPs for joint home visits together with the community nurse to newly discharged COPD patients [40].

Targeted self-management support for patients to cope with exacerbations of the disease was an integral part of our strategy, and we developed an action card with advice to patients on management of sputum and exacerbations. The action card was based on the research by Robert Stockley [41,42].

To provide family, friends and the patients themselves with more knowledge to improve their ability to cope with their disease, we designed a web site with information about COPD including contact details to the municipality, patient support groups and the involved GPs.

The standard implementation of the disease management programme from the Central Denmark Region went ahead and thus also covered all the groups in our study.

### Randomisation and sample size

#### Randomisation and allocation concealment

All GP practices were included in the study. An independent researcher drew slips that were matched to an electronic record of all GP practices in the Ringkoebing-Skjern Municipality. The practices were block-randomised into three blocks. The first block consisted of solo-practices; three practices were drawn to the intervention group and two to the control group. The second block consisted of practices with two GPs; three practices were drawn to the intervention group and two to the control group. The third block consisted of practices with three or more GPs; three practices were allocated to the intervention group and three to the control group. In the external control group, there were two solo-practices, three practices with two GPs and four with three or more GPs. One practice with three GPs was allocated to the intervention group as one of the GPs was partly involved in the overall planning of the study. Out of the nine invited intervention practices, seven accepted the invitation to participate. In accordance with the intention-to-treat principle, the two practices that declined the invitation to participate remained in the intervention group. In total, 21 GPs were randomised to the intervention group and 17 to the control group; the external control group consisted of 25 GPs. The characteristics of the GPs can be found in Table 1.

**Table 1:** Baseline characteristics for GPs as of 2008

	**Intervention**	**Control**	**Ext. control**	**Total**
**N (%)**	21 (33.3)	17 (27.0)	25 (39.7)	63 (100)
**Male (%)**	14 (66.7)	13 (76.5)	13 (52.0)	40 (63.5)
Mean age in years (min-max)*	51.6 (36–62)	49.2 (37–65)	51.6 (32–63)	50.9 (32–65)
Mean number of patients per GP (SD) *	1579 (210)	1290 (457)	1108 (295)	1296 (386)
No COPD CME^** ^**Attendance (%)**	4 (21.1)	5 (38.5)	#	#
**Did not routinely perform spirometry for smokers with symptoms (%)**	10 (50.0)	4 (30.8)	#	#
**Did not routinely stratify patients with COPD (%)**	9 (45.0)	6 (46.2)	#	#
**Did not routinely note the MRC score in the patient file (%)**	15 (75.0)	10 (76.1)	#	#
**Did not routinely provide prescription for antibiotics (%)**	18 (90.0)	11 (84.6)	#	#

*p<0.001 Chi square test or linear regression model applied.

**CME COPD-related continued education.

#No questionnaire data from external control group.

The allocation of both GPs and patients to the intervention and the control group was open and known to the GPs and also to the researchers as it was not possible to hide the allocation to the researchers who also delivered the intervention to the GP practices.

Patients identified by the COPD algorithm from all three groups were sent a baseline questionnaire and patients from the intervention group were also sent a flyer. Furthermore, a poster was displayed in the practice premises informing the patients that this practice was an intervention practice. Only people coming into the premises could see it. In Denmark, every citizen is listed with a particular GP practice and can only use the services from this practice, and patients rarely want to change practice. It is most unlikely that a person would change practice because of a poster.

#### Sample size

To detect a change from 50 to 60% in the proportion of patients having a yearly follow-up consultation for their chronic disease (with 80% power at the 0.05 level of significance), a total of 816 patients with 408 in each group would have to be included in the study. With a design effect of 1.6, we would need 1,306 patients for the study.

#### The COPD algorithm

The COPD algorithm was validated in a previous study which suggested that it could be used as a tool to identify patients with obstructive lung disease, primarily COPD [31]. The search algorithm was based on administrative data on hospitalisation, redeemed prescriptions for lung-related drugs and spirometries performed in the GP practice; thus, the patients had already been in contact with the healthcare system for a lung-related issue. A prerequisite for identification was that the patient should be 35 years old or older and be registered with a GP practice in the patient’s residing municipality. The patients were identified either because they had been hospitalised during the past five years with a lung-related diagnosis, had redeemed prescriptions on lung medication at least twice during the past year or had had their lung function tested at their GP on two different occasions during the past year.

#### Participants

**Figure 1:**
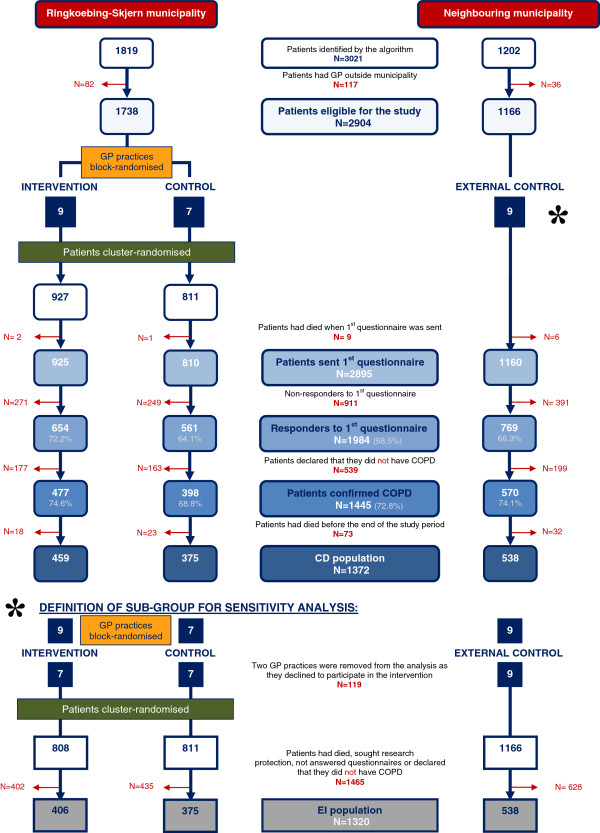
**Flowchart for cluster-randomised study and patient flow in this and in the extra added group.** Patient inclusion and exclusion presented in numbers with percentage distribution indicated below numbers.

The patient population comprised 3,021 patients from the two municipalities who were identified by the COPD algorithm [31]. Among these patients, 1,819 were from the Ringkoebing-Skjern Municipality and 1,202 were from the neighbouring municipality. Of the identified patients, 2,895 had a GP in the municipality of their residence. At follow-up, 159 had died or sought research protection. All patients received a questionnaire at baseline. The study population consisted of responders who confirmed their COPD diagnosis, called the CD population. Patients who died during the intervention period were excluded (Figure 1).

All patients followed their GPs’ allocation to intervention, control group or external control group and would only drop out of the study if their GP did so or if they moved out of the municipality. No GP dropped out of the study.

### Data

The primary outcome was adherence to the disease management programme in the GP practices. Adherence was measured at the patient level in terms of use of the specific services of planned and additional preventive consultations, and the number of spirometries. The GP practices report each of these services to the region to be reimbursed.

The secondary outcome was use of out-of-hours services, outpatient clinic and emergency department attendances as well as hospital admissions. For admissions to hospitals, we defined a readmission as an acute admission concerning COPD within 30 days of the last admission.

#### Register data

We collected data from the DNHISR [33,34] and the regional PAS for all 2,736 patients identified by the COPD algorithm from 1 November 2008 to 31 October 2010 (12 months before and 12 months after the start of the intervention).

#### Questionnaire data

A baseline questionnaire was sent to all identified patients. The questionnaire included validated tools to assess the patients’ respiratory state and mental health; their experience of their health, care and practice (Medical Research Council’s dyspnoea scale [43], the Major Depression Inventory (MDI) [44], their self-reported health (EQ-5D) [45], the Patient Assessment of Chronic Illness Care (PACIC) [46] and the Danish Patients Evaluate Practice (DanPEP) [47]). The questionnaire also contained questions regarding use of medication, support and socio-economic issues developed on the basis of literature, clinical experience and interviews with patients and healthcare professionals. Patients who confirmed their diagnosis were sent a questionnaire at follow-up. The results from the PACIC have been reported elsewhere [48].

### Analyses

The effect of the active implementation was analysed by comparing the changes in the intervention group with the changes in both the control group and the external control group. For each of the outcomes, we calculated yearly rates and rate ratios (RRs) between the year before and the year after the intervention. To determine the differences between the groups, the corresponding pairwise RRs between the groups were calculated. To facilitate estimation of the RRs, we used a binomial regression model with log link when analysing each of the following outcomes: proportion of planned preventive consultations, additional preventive consultations and performed spirometries, as well as the proportion of patients who had contact to the out-of-hours services, who were admitted with or without a lung-related diagnosis and who had contact to the emergency department. A negative binomial regression model allowing for the heterogeneity between subjects [49] was used to analyse the counts of the following outcomes: conventional consultations, contacts to the out-of-hours services, use of bed days, contacts to the outpatient services with or without a lung-related diagnosis, number of contacts to the emergency department, number of admissions with or without a lung-related diagnosis and number of readmissions. Confidence intervals (CIs) at 95% were assessed by performing robust variance estimation to account for a cluster effect at the GP level and, consequently, also at the patient level. Adjustment was made for age and gender, although this had minimal influence on the estimates of interest.

We performed intention-to-treat analysis for the algorithm-identified population and for the CD population who had been identified by the algorithm and who had themselves confirmed their diagnosis.

To examine the effectiveness and efficiency of the active implementation model, we performed an additional sensibility analysis where the two non-participating practices were omitted from the analysis and practices actually taking part in the intervention were analysed as intervention practices; we called the sub-group with this distribution of patients the EI population.

Analyses were performed using STATA version 11.0. The trial was performed in accordance with the CONSORT statement extended for cluster-randomised controlled trials [50].

### Ethics

The study was recommended by the Committee of Multipractice Studies in General Practice under the Danish College of General Practitioners and by the Organisation of General Practitioners in Denmark (MPU 17–2009). The study was approved by the Danish Data Protection Agency (J.nr. 2008-41-2855) and the Danish Health and Medicines Authority (J.nr. 7-604-04-2/71 /EHE). The RCT was indexed at http://www.clinicaltrials.gov/show/NCT01228708 (ID number: NCT01228708). The present project did not require approval under the Danish health research ethics committee system (the Committee Act).

## Results

### Patients

The patients identified by the algorithm and those patients who also confirmed their COPD diagnosis, i.e. the CD group, are characterised in Table 2. In the CD group, the intervention group counted 458 (33.4%) patients, the control group 376 (27.4%) patients and the external control group 538 (39.2%) patients. The EI population comprised 406 (29.6 %) patients in the intervention group, 376 (27.4%) patients in the control group and 538 (39.2%) patients in the external control group (Figure 1).

**Table 2:** Baseline data for patients as listed in the Danish Health Insurance Service Registry by 1 November 2008

**Patients identified by COPD algorithm**				
	**Intervention**	**Control**	**Ext. control**	**Total**
**N (%)**	877 (32.1)	766 (28.0)	1,092 (39.9)	2,735 (100)
**Men (%)**	399 (45.5)	354 (46.2)	492 (45.0)	1,244 (45.5)
**Female (%)**	478 (54.5)	412 (53.8)	600 (55.0)	1,491 (54.5)
**Mean age (min-max)**	63.9 (35–97)	63.3 (35–96)	63.3 (35–101)	63.5 (35–101)
**CD Population**				
**Patients identified by algorithm who confirmed that they had COPD**				
	**Intervention**	**Control**	**Ext. control**	**Total**
**n (%)**	458 (33.4)	376 (27.4)	538 (39.2)	1,372 (100)
**Proportion of N (%)**	52.2	49.1	49.3	50.2
**Mean age (min-max)**	222 (48.5)	179 (47.6)	264 (49.1)	665 (48.5)
**Men (%)**	236 (51.5)	197 (52.4)	274 (50.9)	707(51.5)
**Female (%)**	67.6 (36–91)	66.3 (35–91)	66.7 (36–94)	66.9 (35–94)

### Primary outcome

Tables 3 and 4 show the changes in each group and the differences in changes between the groups for the study population. Data from the sub-group analysis are not shown.

**Table 3:** Rates and rate ratios (RRs) for specific variables presented with 95% confidence intervals

**N= 1372**		**Intervention (N=458)**	**Control (N=376)**	**Ext. control (N=538)**	**Intervention vs. Control RR**	**Intervention vs. Ext control RR**	**Control vs. Ext control RR**
**Number of patients who had a planned preventive**	2008-09	**0.34** 0.27;0.44	**0.24** 0.17;0.33	**0.25** 0.14;0.45	**1.43** 0.96;2.13	**1.35** 0.72;2.52	**0.95** 0.49;1.81
**consultation**	2009-10	**0.53** 0.42;0.68	**0.21** 0.15;0.30	**0.29** 0.17;0.48	**2.52**^**1**^ 1.62;3.92	**1.84**^**1**^ 1.05;3.24	**0.73** 0.39;1.36
	**RR**	**1.55**^**2**^ 1.23;1.96	**0.88** 0.69;1.12	**1.14** 0.91;1.42	**1.77**^**2**^ 1.26;2.48	**1.36** 0.98;1.88	**0.77** 0.55;1.07
**Number of patients who had an additional **	2008-09	**0.11** 0.05;0.27	**0.06** 0.03;0.14	**0.10** 0.06;0.18	**1.86** 0.56;6.17	**1.09** 0.39;3.07	**0.59** 0.22;1.55
**preventive consultation**	2009-10	**0.23** 0.11;0.48	**0.08** 0.03;0.21	**0.12** 0.07;0.24	**3.00** 0.87;10.35	**1.86** 0.70;4.91	**0.62** 0.19;2.03
	**RR**	**2.03**^**2**^ 1.26;3.27	**1.26** 0.84;1.89	**1.19** 0.84;1.71	**1.61** 0.86;3.01	**1.70** 0.94;3.09	**1.05** 0.62;1.81
**Number of patients who had a spirometry**	2008-09	**0.33** 0.26;0.41	**0.23** 0.16;0.33	**0.22** 0.17;0.28	**1.43** 0.92;2.23	**1.48**^**1**^ 1.05;2.08	**1.03** 0.66;1.63
**performed at the GP practice**	2009-10	**0.45** 0.34;0.58	**0.24** 0.16;0.36	**0.22** 0.15;0.34	**1.82**^**1**^ 1.23;2.94	**2.01**^**1**^ 1.23;3.01	**1.10** 0.62;1.96
	**RR**	**1.36**^**1**^ 1.09;1.70	**1.07** 0.85;1.34	**1.00** 0.70;1.44	**1.27** 0.93;1.75	**1.36** 0.89;2.08	**1.07** 0.70;1.64
**Number of patients who were admitted to hospital**	2008-09	**0.24** 0.20;0.29	**0.21** 0.17;0.26	**0.24** 0.21;0.28	**1.16** 0.86;1.55	**1.01** 0.80;1.29	**0.88** 0.67;1.15
**without a lung-related diagnosis**	2009-10	**0.22** 0.18;0.26	**0.26** 0.23;0.30	**0.26** 0.21;0.31	**0.82** 0.66;1.02	**0.83** 0.64;1.07	**1.01** 0.81;1.26
	**RR**	**0.88** 0.74;1.05	**1.24** 0.99;1.56	**1.08** 0.92;1.26	**0.71**^**2**^ 0.53;0.94	**0.82** 0.65;1.03	**1.15** 0.87;1.52

^1^Statistical significance p ≤ 0.05.

^2^Statistical significance p ≤ 0.005.

No noteworthy difference was found for the proportion admitted with a lung-related diagnosis. Data not shown.

**Table 4:** Counts and incidence rate ratios (IRRs) for specific variables presented with 95% confidence intervals

**N= 1372**		**Intervention (N=458)**	**Control (N=376)**	**Ext. control (N=538)**	**Intervention vs. Control IRR**	**Intervention vs. Ext control IRR**	**Control vs. Ext control IRR**
**Consultations with GP (daytime)**	2008-09	**7.75** 6.76-8.87	**7.57** 6.43-8.92	**6.75** 6.24-7.29	**1.02** 0.83-1.26	**1.15** 0.98-1.34	**1.12** 0.94-1.34
	2009-10	**7.34** 6.60-8.17	**8.50** 7.12-10.14	**7.28** 6.73-7.88	**0.86** 0.71-1.06	**1.01** 0.88-1.15	**1.17** 0.96-1.41
	**IRR**	**0.95** 0.88-1.02	**1.12**^**2**^ 1.08-1.17	**1.08**^**2**^ 1.02-1.44	**0.85**^**2**^ 0.78-0.92	**0.88**^**1**^ 0.80-0.96	**1.04** 0.97-1.12
**Contacts to out-of-hours services**	2008-09	**0.68** 0.47-0.98	**0.60** 0.42-0.86	**0.61** 0.51-0.74	**1.12** 0.66-1.90	**1.10** 0.73-1.66	**0.99** 0.66-1.47
	2009-10	**0.69** 0.47-1.01	**0.74** 0.56-0.98	**0.56** 0.46-0.68	**0.93** 0.60-1.45	**1.23** 0.83-1.83	**1.32** 0.94-1.85
	**IRR**	**1.02** 0.85-1.22	**1.22**^**1**^ 1.02-1.48	**0.92** 0.78-1.08	**0.83** 0.64-1.08	**1.12** 0.88-1.42	**1.34**^**1**^ 1.05-1.71
**Admissions with another diagnosis**	2008-09	**0.35** 0.27-0.45	**0.36** 0.28-0.46	**0.33** 0.28-0.38	**0.97** 0.68-1.38	**1.06** 0.79-1.43	**1.10** 0.82-1.46
	2009-10	**0.33** 0.27-0.41	**0.47** 0.38-0.58	**0.45** 0.36-0.56	**0.70**^**1**^ 0.52-0.94	**0.74** 0.55-1.01	**1.06** 0.78-1.43
	**IRR**	**0.95** 0.69-1.32	**1.31** 0.91-1.88	**1.36**^**1**^ 1.06-1.74	**0.73** 0.44-1.18	**0.70** 0.47-1.05	**0.96** 0.62-1.49
**Bed days**	2008-09	**1.56** 1.09-2.22	**2.07** 1.55-2.63	**1.48** 1.11-1.98	**0.77** 0.49-1.21	**1.05** 0.67-1.63	**1.36** 0.90-2.06
	2009-10	**1.61** 1.33-1.96	**1.70** 1.26-2.29	**2.01** 1.59-2.56	**0.95** 0.66-1.36	**0.80** 0.60-1.07	**0.84** 0.58-1.23
	**IRR**	**1.04** 0.69-1.56	**0.84** 0.64-1.11	**1.36**^**1**^ 1.09-1.70	**1.23** 0.75-2.02	**0.76** 0.48-1.21	**0.62**^**1**^ 0.43-0.89
**Readmissions^**	2008-09	**0.07** 0.05-0.12	**0.13** 0.10-0.17	**0.05** 0.04-0.08	**0.56**^**1**^ 0.33-0.97	**1.38** 0.78-2.47	**2.45**^**2**^ 1.53-3.94
	2009-10	**0.07** 0.05-0.09	**0.12** 0.06-0.21	**0.14** 0.11-0.19	**0.59** 0.30-1.15	**0.48**^**1**^ 0.32-0.72	**0.82** 0.42-1.60
	**IRR**	**0.95** 0.55-1.63	**0.90** 0.50-1.64	**2.71**^**2**^ 1.90-3.86	**1.05** 0.46-2.38	**0.35**^**2**^ 0.18-0.66	**0.33**^**2**^ 0.16-0.68
**Contacts to outpatient services with any**	2008-09	**3.07** 2.70-3.48	**3.38** 2.83-4.03	**3.03** 2.59-3.54	**0.91** 0.73-1.32	**1.01** 0.84-1.22	**1.12** 0.88-1.42
**diagnosis**	2009-10	**2.91** 2.55-3.34	**3.68** 3.30-4.11	**3.65** 2.96-4.49	**0.79**^**2**^ 0.67-0.94	**0.80** 0.63-1.01	**1.01** 0.80-1.27
	**IRR**	**0.95** 0.81-1.11	**1.09** 0.95-1.25	**1.20**^**2**^ 1.22-1.29	**0.87** 0.71-1.07	**0.78**^**1**^ 0.66-0.94	**0.90** 0.78-1.04
**Contacts to outpatient services with**	2008-09	**0.16** 0.11-0.24	**0.28** 0.19-0.40	**0.19** 0.12:0.30	**0.59**^**1**^ 0.35-0.99	**0.86** 0.48-1.57	**1.47** 0.83-2.62
**a lung-related diagnosis **	2009-10	**0.22** 0.13:0.36	**0.28** 0.17-0.46	**0.26** 0.19-0.38	**0.78** 0.67-0.94	**0.83** 0.45-1.54	**1.07** 0.59-1.92
	**IRR**	**1.35** 0.76-2.40	**1.02** 0.67-1.54	**1.41**^**2**^ 1.13-1.74	**1.33** 0.65-2.70	**0.95** 0.51-1.81	**0.72** 0.46-1.15

^A readmission was defined as an acute admission within 30 days of the last admission.

^1^Statistical significance p < 0.05.

^2^Statistical significance p < 0.005.

No noteworthy difference was found for the admissions with a lung-related diagnosis. Data not shown.

The number of patients who had a planned preventive consultation rose statistically significantly in the intervention practices compared with the control practices (RR=1.77, p=0.001). Intervention patients doubled their additional preventive consultations (RR=2.03, p=0.004) and more had a spirometry at least once a year (RR=1.36, p=0.006).

Interestingly, the use of conventional consultations decreased among the patients in the intervention group, while an increase was seen in the control group; thus, there was a statistically significant difference in use of conventional consultations (RR=0.85, p=0.005).

### Secondary outcome

Patients from the control group made more contacts to the out-of-hours services at the end of the study period than in the beginning (IRR=1.22, p=0.032). No difference in the change of contacts to out-of-hours services was observed between the intervention group and the control groups.

Fewer patients from the intervention group were admitted without a lung-related diagnosis than in the control group (RR=0.71, p=0.018); and fewer patients from the intervention group were readmitted than in the external control group (IRR=0.33, p=0.003). The use of hospital bed days did not change in the intervention group; whereas it rose in the control group (IRR=1.35, p=0.008).

Although patients from the external control group visited the outpatient services more at the end of the study period (IRR=1.41, p=0.002), there was no difference in the change in visits to the outpatient services between the intervention group and either the control group or the external control group.

No difference was observed in the three groups’ use of emergency department services before and after the intervention.

### Sensitivity analyses

The results of the analysis in which the data from the two practices that did not participate in the intervention were removed were fairly similar to the findings reported above although there were two new findings: more patients in the intervention group than in the control group had an additional preventive consultation (RR=1.95, p=0.049) and the number of acute admissions in the intervention group fell to less than half (RR=0.43, p=0.002) (data not shown). The same was the case for the results for the population identified by the algorithm both for the intention-to-treat and the sensitivity analysis (data not shown). For the population identified by the algorithm, the intention-to-treat analysis showed the same number of readmissions in the intervention group after the active implementation, but the number of readmissions rose in both the control group and the external control group (IRR=1.41, p<0.001; and IRR=1.66, p=0.014).

## Discussion

### Principal findings

Our study showed an effect of the intervention on planned and additional preventive consultations, performed spirometries and admissions. Thus, the intervention changed the management of patients with COPD.

### Strength and limitations of the study

This study draws strength from its randomised design, the high number of included GPs and patients, and the use of highly valid administrative data. The results have been analysed both for the population identified by the COPD algorithm and for the study population where patients had confirmed their diagnosis. It adds to the strength of the study that the study population’s results were either reproduced in the sub-group analysis or even enhanced. A further strength springs from the fact that a strong trend towards the same results was identified in the analysis of the population identified by the algorithm alone.

The two practices with four GPs who declined to participate in the intervention were excluded in a sub-group analysis because we wanted to examine the effectiveness of the active implementation model. These two practices received none of the components of the active implementation. This could give rise to selection bias. However, the sub-group analysis showed an overall pattern very close to that of the intention-to-treat analysis, which assured us that this bias is not decisive.

In order to accommodate analyse changes over time we chose to exclude those patients who died during the trial. In case of large differences in mortality between groups, this could affect the results of the comparison of health care use in either direction. However, the nature of the intervention and the nature of the relatively slowly evolving disease convinced us that any influence of the intervention on the endpoint “death” would be negligible in a short-term perspective. This assumption was confirmed by the present data.

We did consider if research protection could be an issue for patients in the randomised groups. One could think that patients might tire of being contacted. Both the intervention group and the control group were exposed to the baseline and the follow-up questionnaire, but only intervention patients received a flyer with the questionnaire; this would hardly trigger an action to seek research protection.The present data show that the issue of research protection played only a negligible role for participation.

The use of a complex intervention [35] building on specific aspects of the CCM [3,4] represented a special challenge. For example, we were unable to assess the effectiveness of the individual components of the intervention. The implementation was offered as a package and was evaluated as such even though each individual GP practice may have selected only particular elements from the full catalogue. This may have introduced variation as to which elements actually brought change. However, in these respects, the present implementation processes were in no way different from similar implementation processes in the healthcare system.

Despite our efforts to identify as many of the patients with known COPD as possible, our study is underpowered. We were only able to identify those patients who had been in contact with the healthcare system and for whom lung-related complaints had resulted in an action on the part of the healthcare system. When a method to detect COPD in the earlier stages has been developed, patients with milder degrees of COPD can be included in the analyses. This will allow us to conduct a study of a larger scale; alternatively, the study can be extended to more municipalities participating in the randomised study.

An independent person randomised GP practices to the intervention or the control group. However, after the randomisation, we could not conceal the allocation to the researchers who performed the analysis because this group of researchers was also the one which conducted the intervention with the intervention practices. This could be considered a flaw in the study design.

One weakness of the present intervention, which is shared by most health services research, is that some intervention elements (e.g. recruitment of patients for the courses) were available for patients from the entire Ringkoebing-Skjern Municipality and not exclusively for patients from the intervention practices. This may lead to a serious underestimation of the effect and could imply that we have found the least possible difference between the study groups.

The understanding of the intervention could have been enhanced by including a process evaluation component into the study. For example, we could have interviewed the patients and/or the health professionals.

The added external control group further strengthened the study because it allowed us to check the spillover effect within the CME groups and in the common health services for all COPD patients.

### Comparison with other studies

Other studies have shown similar effects of targeted, planned and evidence-based interventions for patients with chronic conditions [51-53] even when the impact was measured after ten years [54], whereas others have found no effects [55]. One meta-analysis suggests that comparing implementation strategies may establish sound evidence for the effectiveness of the successful implementation of programmes [56].

In a systematic review of the use of the CCM for COPD prevention and management, Adams et al. drew the conclusion that patients with COPD who received two or more components of the CCM had fewer unscheduled/emergency-room visits, fewer hospitalisations and showed a trend towards reduced healthcare costs compared with the control groups [57]. We made a similar observation, even if the effect on the use of the emergency department visits was minimal and we used healthcare utilization as a proxy for healthcare costs.

The intervention with the components that we selected is directly transferrable and applicable in future multifaceted interventions for chronic conditions in general practice.

Our finding of less use of hospital services after active implementation of a disease management programme is highly relevant as other studies have shown an increase of 2.3 in hospital admission rates for COPD patients compared with patients without COPD [58].

### Unanswered questions and future research

The study period was two years with one year before the intervention start and one year after. The effect may have been different if the study had covered a longer period of time.

We measured healthcare utilization as a proxy for economic evaluation. A detailed and more comprehensive analysis, including the costs of the implementation, is needed. Consideration of the economic aspects of the active implementation of optimised healthcare for patients with COPD would also be of future interest.

The study would benefit from a deeper look into the black box of the intervention. An added process evaluation component could provide such insight as would also a future study with interviews with patients and/or with healthcare professionals. Another future study could include the component in a mixed methods study.

Multimorbidity is often the norm in general practice. A strategy like the implementation model used in the present study may be an effective tool to coordinate healthcare services for patients with multimorbidity. A future study including patients with multimorbidity would be of much interest to clinicians and politicians.

Our findings in this randomised study and the success of similar programmes for other chronic diseases highlight the potential and the need for larger long-term studies. Such studies should explore activities and implementation strategies that could easily be adopted in GP practices and should comprise multiple components of the CCM with a view to preventing complications and improving outcomes for patients with COPD.

## Conclusions

The multifaceted implementation of a disease management programme for COPD made general practice follow the programme and proactively perform follow-up consultations and spirometries. This resulted in a decline in the number of GP consultations and a tendency towards decreased use of hospital services. Use of out-of-hours services remained the same although patients from the control group increased their use of these services. No effect on the use of the emergency department was found. The implementation model can be applied in future multifaceted interventions targeting patients with chronic disease.

## Abbreviations

AT: As treated; CCM: Chronic Care Model; CD: Confirmed Diagnosis; CI: Confidence Interval; COPD: Chronic Obstructive Pulmonary Disease; GP: General Practitioner.

## Competing interests

The authors declare that they have no competing interests.

## Authors' contributions

FO, PV and MS designed the study. MS, PV, FO and MBC participated in the study and in drafting the manuscript. MFG assisted with statistical analysis and in drafting the manuscript. All authors have read and approved the final manuscript.

## Pre-publication history

The pre-publication history for this paper can be accessed here:

http://www.biomedcentral.com/1472-6963/13/385/prepub

## Supplementary Material

Additional file 1 **The PaTPlot depicting the timeline and the contents of the active implementation model.** Squares illustrate fixed object, e.g. printed materials like questionnaires. Circles illustrate an activity was involved in the component, e.g. Continuing Medical Education meetings. Click here for file
